# Engineering the Side Facets of Vertical [100] Oriented InP Nanowires for Novel Radial Heterostructures

**DOI:** 10.1186/s11671-019-3177-6

**Published:** 2019-12-30

**Authors:** H. Aruni Fonseka, Philippe Caroff, Yanan Guo, Ana M. Sanchez, Hark Hoe Tan, Chennupati Jagadish

**Affiliations:** 1 Department of Electronic Materials Engineering, Research School of Physics and Engineering, The Australian National University, Canberra, ACT 2601 Australia; 2 Department of Physics, University of Warwick, Coventry, CV4 7AL UK; 3 Current Address: Microsoft Station Q, Delft University of Technology, 2600 GA Delft, Netherlands; 4 Current Address: Samsung Austin Semiconductors, 12100 Samsung Blvd, Austin, TX 78754 USA

**Keywords:** [100] oriented nanowires, Nanowire facets, Cross-section shape, Four-sided radial heterostructures

## Abstract

In addition to being grown on industry-standard orientation, vertical [100] oriented nanowires present novel families of facets and related cross-sectional shapes. These nanowires are engineered to achieve a number of facet combinations and cross-sectional shapes, by varying their growth parameters within ranges that facilitate vertical growth. In situ post-growth annealing technique is used to realise other combinations that are unattainable solely using growth parameters. Two examples of possible novel radial heterostructures grown on these vertical [100] oriented nanowire facets are presented, demonstrating their potential in future applications.

## Introduction

Large surface area is one of the key advantages of nanowires compared to conventional thin films. This greatly increases the importance of the nanowire side facets that form those surfaces. Nanowire side facets play an important role in controlling their morphological, structural, electrical, thermal and optical properties [1–5]. Radial nanowire heterostructures are directly linked to facets that they are grown on. Uniform nanowire side facets such as {0–11} mostly yield uniform radial heterostructures in [111] oriented nanowires [6, 7]. On the other hand, growth on facets that are non-uniform, either in terms of crystal plane, polarity or dimensions can be used to create complex radial structures such as nanocavities, quantum wells with novel geometries, twinned superlattice nanotubes and quantum wires [8–14]. Different surface recombination velocities and nano-scale roughnesses of different facet types affect carrier recombination and phonon transport in nanowires [3, 15, 16]. The nanowire cross-section shape, which is determined by the type of facets and their relative dimensions, is important in applications where the nanowire is used as an optical cavity, as it can affect the types and number of modes that are confined [17–19]. Furthermore, nanowire facets can be used as alternative templates to patterned substrates in order to grow quantum wires and wells, thereby eliminating the need for complex processing and patterning.

Non-nitride III–V nanowires are generally grown on (111) surfaces, due to the ease of achieving vertical [111] oriented nanowires. In case of InP, growth on (111) substrates commonly yield wurtzite (WZ) phase nanowires or zincblende (ZB) twin super-lattices [20, 21], with resulting facet profiles consisting of {1-100}, {11-20} or {111} type facets. Cross-sectional shapes are mostly of hexagonal or truncated triangular shape. Change of growth orientation can be used as a key method to demonstrate unconventional side facets combinations and cross-section shapes [22, 23]. In addition to being grown on the industry-standard substrate orientation and being defect-free ZB [24, 25], <100> nanowires open up a completely new families of available facets, their combinations and resulting cross-sectional shapes, such as square and octagonal shapes, that are difficult to be obtained in nanowires grown in other orientations [22–24]. These facets and their combinations that are not well studied so far could open up many possibilities in terms of applications of nanowire facets discussed above.

In this work, the facets of the [100] oriented InP nanowires are engineered to achieve different types of facets and varying degrees of their combinations, by which a number of resulting cross-section shapes are realised. The novel cross-sectional shapes include square, rectangle, elongated hexagon, elongated octagon and perfect octagon. All combinations discussed are demonstrated while maintaining a high yield of vertical [100] nanowire growth, using the techniques discussed in [24] and [26], which enhances their ability to be used in applications. First, the effects of growth conditions on the resulting facets are discussed in order to gain an understanding of their relative formation. Next, post-growth in situ annealing of the nanowires is used as a technique to further achieve novel combinations of facets that are not achievable simply by tuning growth parameters which are restricted by the stringent requirements for the vertical [100] nanowire growth. The understanding of the relationship between the relative growth of facets and respective growth conditions is used to achieve selective growth only on some of the nanowire facets and hence form four-sided, partitioned nanowire radial heterostructures.

## Methods

Nanowires were grown using a horizontal flow metal-organic vapour phase epitaxy (MOVPE) reactor with a total flow rate of 15 slm, using TMIn and PH_3_ as precursors. Two separate pre-growth conditions that have been previously reported to yield a high percentage [100] vertical nanowires on [100] oriented InP substrates were used [24, 26] (here, the vertical yield defined as the percentage of catalyst particles in a sample area that results in [100] vertical nanowires). The colloidal Au particles were deposited on the substrates with the aid of a poly-L-lysine layer. In the first method (*pre-growth condition 1*), the substrates were annealed at 450 °C under a PH_3_ flow of 8.93 × 10^−4^ mol/min for 10 min before initiating the growth at the same temperature [24]. 30 nm Au particles were used as seed particles in this study due to this size yielding the highest percentage of vertical nanowires for the *pre-growth condition 1* specified above. In the second method (*pre-growth condition 2*), instead of annealing, TMIn was pre-flown for 15 s after ramping the temperature to the growth temperature of 450 °C [26]. 50 nm Au particles were used in this study, as the *pre-growth conditions 2* had been optimised for this particle size [26, 27]. Growths which used *pre-growth conditions 1*, were based around nanowire growth conditions shown in Table 1, where the specified parameter was varied while others were kept constant. For the higher TMIn flow rate growths the growth time was reduced in order to keep the nanowire dimensions comparable.

**Table 1:** Growth parameters of the standard sample using *pre-growth conditions 1*

Growth temperature	450 °C
V/III precursor flow rate ratio (in vapour phase)	350
TMIn flow rate for the nanowire growth	2.02 × 10^−6^ mol/min
Nanowire growth time	1 hr

The nanowires grown using *pre-growth condition 2* were grown using parameters shown in Table 2. For the growths where the TMIn flow rate was increased by three times, the TMIn pre-flow and nanowire growth times were reduced proportionately.

**Table 2:** Growth parameters of the standard sample using *pre-growth conditions 2*

Growth temperature	450 °C
V/III precursor flow rate ratio (in vapour phase)	309
TMIn flow rate for the nanowire growth	1.62 × 10^−5^ mol/min
Nanowire growth time	30 min

Morphological analysis was carried out using Zeiss Ultra Plus and FEI Helios 600 NanoLab Scanning electronic microscope (SEM) while transmission electron microscope (TEM) analysis was carried out using JEOL 2100 TEMs operating at 200 kV. Cross-sections of the nanowire radial heterostructures were prepared by microtome slicing. Photoluminescence (PL) was collected by exciting single nanowires that were spread on a sapphire substrate using a 633 nm HeNe laser with a spot size of ~ 1 μm. Excitation power was 20 μW and the PL was detected by a nitrogen cooled InGaAs detector.

## Results and Discussion

**Fig. 1:**
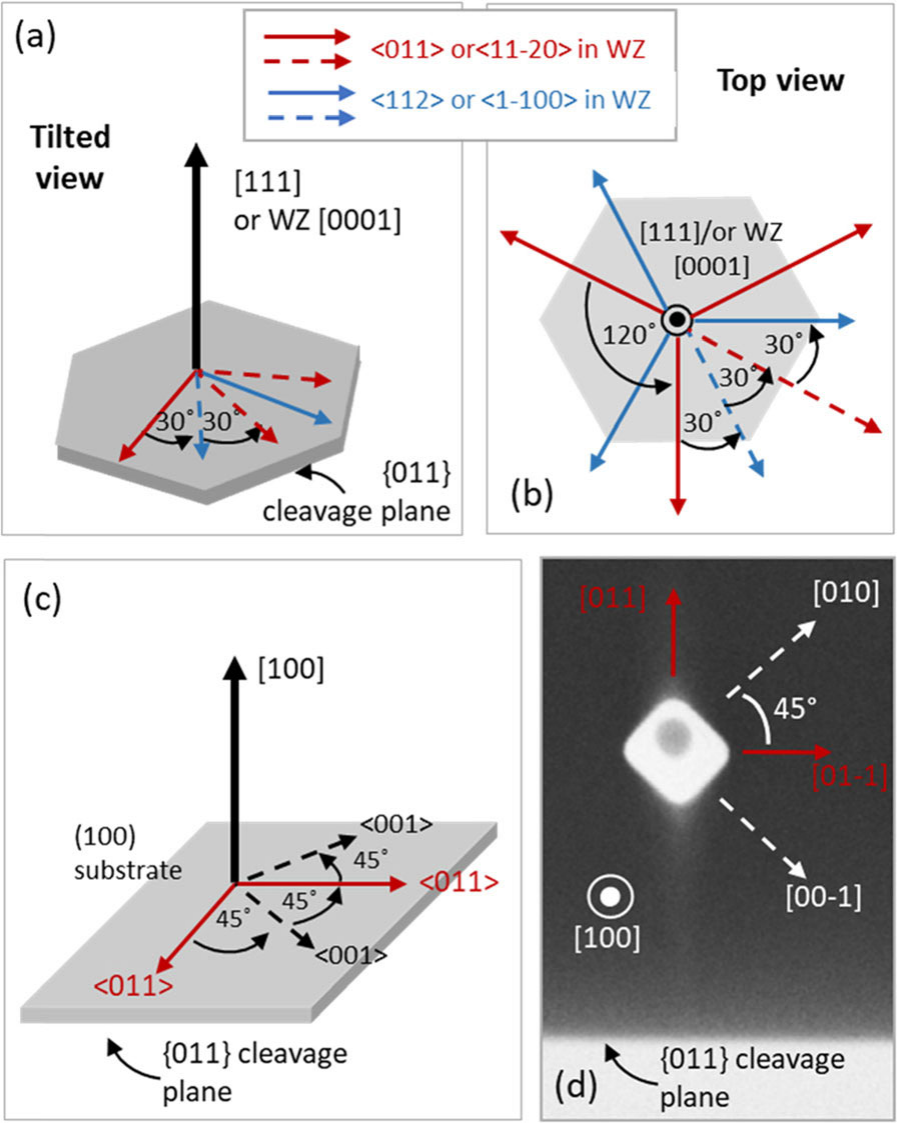
Relative directions of facets in [111] (or WZ [0001]) and [100] oriented nanowires, (**a**) Tilted view of relative directions on the (111) surface. (**b**) Top view of relative directions on the (111) surface. (**c**) Tilted view of relative directions on the (100) surface. (**d**) Top view scanning electron microscopy (SEM) image of a [100] nanowire and the {011} cleavage plane of the (100) InP substrate. Relative directions perpendicular to the facets are indicated.

Nanowire facets generally tend to take the low index and low energy planes that are parallel to their growth direction. In the case of conventional nanowires grown on (111) substrates, {0-11} and {11-2} side facets (or their WZ equivalent {1-100} and {11-20}facets) are most commonly observed, yielding hexagonal, triangular or combinational cross-sectional shapes such as nonagonal and dodecagonal [22, 28]. Figure 1a, b show the tilted and top view of the directions perpendicular to these facets with respect to the nanowire growth direction and (111) substrate. In some cases, such as in {11-2} facets, even though the actual micro-planes are not parallel to the growth direction, the combination of such planes form a resultant plane that is parallel to the growth direction [28].

In the face-centred-cubic (fcc) crystal structure, the low index planes that are parallel to the [100] direction are the {011} and {001} families. Their directions relative to the [100] nanowire growth direction are shown in Fig. 1c. Figure 1d shows a top view SEM image of a nanowire relative to the {011} cleavage plane of the InP substrate, used for easy identification of facets. Table 3 shows the possible combinations and cross-sectional shapes comprising of the aforementioned {011} and {001} low index facets. Facets of both, {011} and {001} families are equivalent and non-polar. However, the {011} surfaces that are slightly off-cut towards [100] nanowire growth direction (as it would be in a tapered nanowire) would show partial polarity, with (01-1) and (0-11) pair of facets showing group V-rich partial B polarity and opposite (011) and (0-1-1) pair of facets showing group III-rich partial A polarity [24]. Under group V-rich, high V/III growth conditions similar to those used in this study, the A polar facets grow faster than B polar facets [29–31]. Similarly, B polar InP surfaces decompose much faster than A polar surfaces due to the two unpaired electrons associated with the P atoms [32, 33]. Although the bonds are not exactly similar in the current case of partial polarity, similar trends in reactivity can be expected due to a higher fraction of P atoms on tilted (01-1) and (0-11) facets. Such anisotropies between these two types of facets make the anisotropic geometry types III, V, VI and VII possible. The two types ((01-1)/(0-11) and (011)/(0-1-1)) can be identified with respect to the <111> non-vertical nanowires grown on the same substrate that takes group V terminated ‘B’ polarity [24].

**Table 3:**
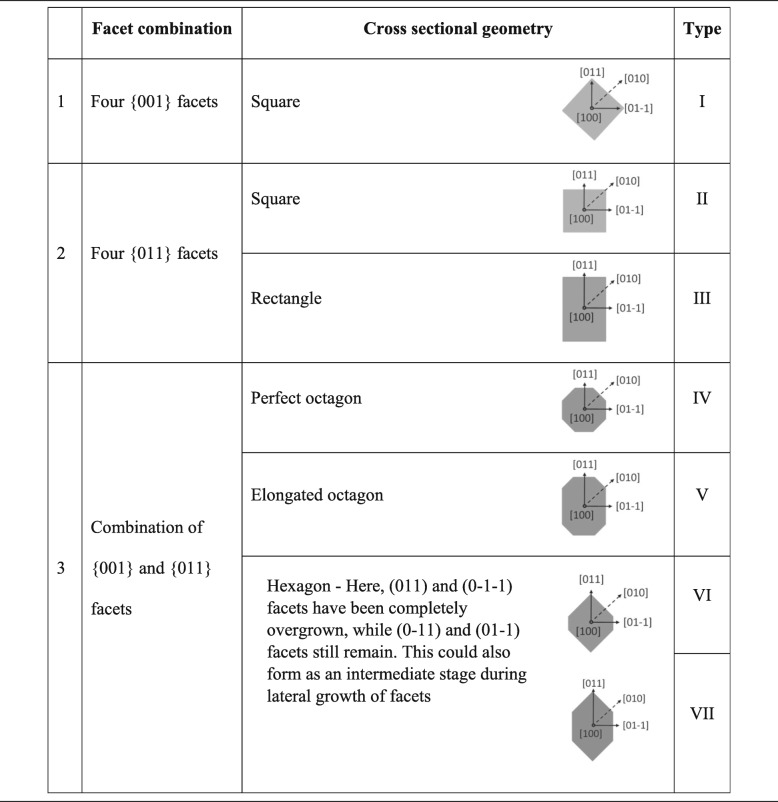
Possible {001} and {011} low index facet combinations and cross sectional shapes for <100> oriented nanowires

It should also be mentioned here that the facets just below the particle form an octagonal shape, which is the polygon shape made up of low index facets that is closest to a circular shape [24]. This in turn allows the particle to remain close to a spherical shape with minimum distortion and surface energy [21, 26]. This work discusses the subsequent stable facets and distinct cross-sectional shapes that evolve later (within about 200 nm from the droplet) and accounts for a large portion of the nanowires. The distinct side facets of nanowires evolve mainly with lateral growth. In addition, surface diffusion and surface evaporation also contribute to this [28, 34]. These factors are limited by the kinetics and thermodynamics that are governed by the growth parameters during nanowire growth [28, 35]. Due to the same reason, the nanowire facets depend only on their actual growth conditions and not on the pre-growth conditions discussed under the methods section.

**Fig 2:**
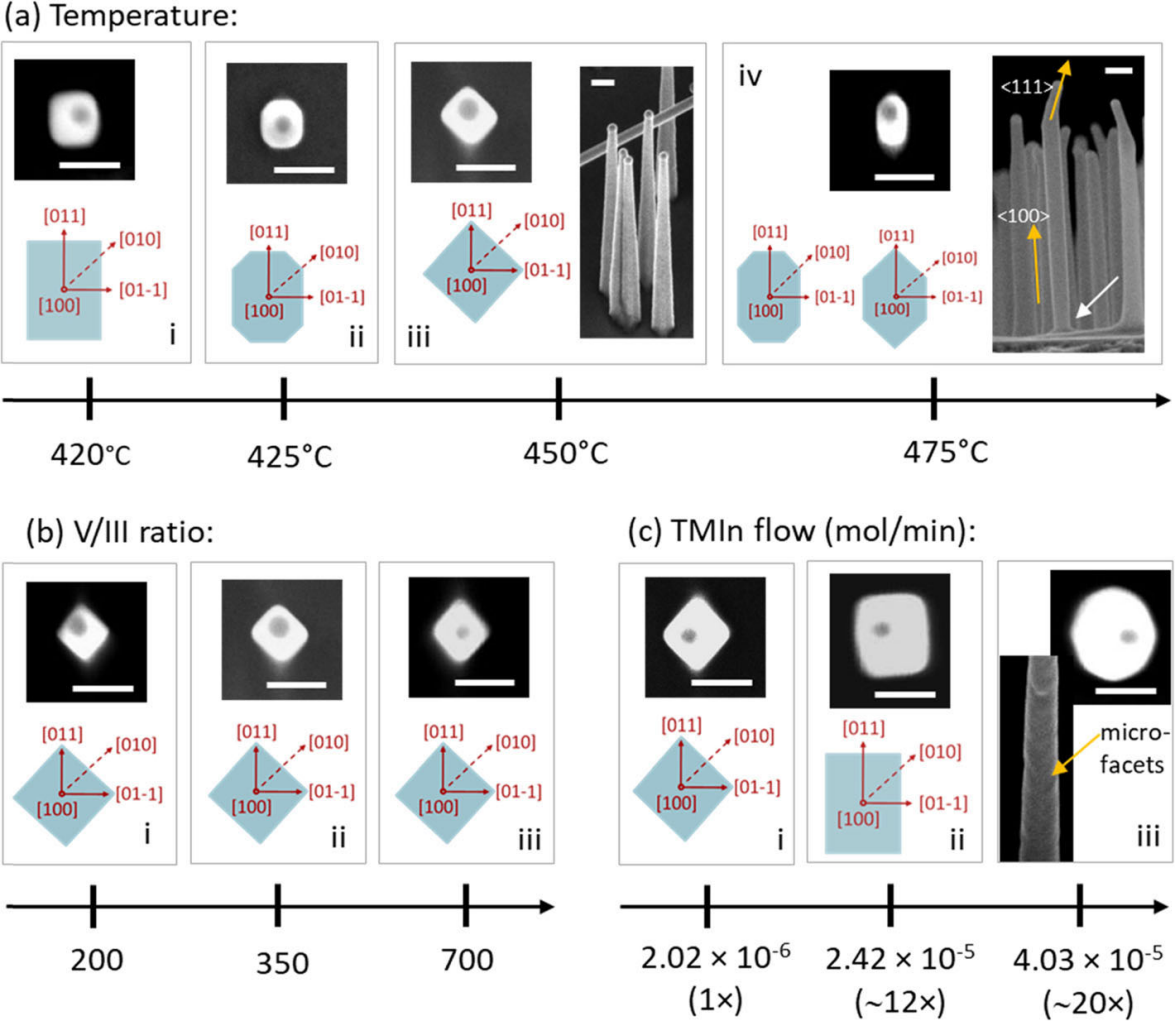
Variation of the side facets of the <100> oriented nanowires with the basic growth parameters. The series along each row correspond to variation in (**a**) growth temperature, (**b**) V/III ratio, (**c**) TMIn flow rate (while keeping V/III constant) with respect to the standard sample grown with growth conditions given in Table 1 in methods section. The white arrow in (**a**)iv indicates the thinner base. Scale bars are 100 nm.

Growth temperature and V/III precursor flow rate ratio are the most influential parameters in MOVPE nanowire growth [35]. In addition to these, the precursor flow rates also affect the growth dynamics [35]. Figure 2a–c shows the facet variation of the [100] oriented nanowires with growth temperature, V/III ratio and trimethylindium (TMIn) flow rate (while keeping V/III constant) during growth. The facet analysis is done using the top view SEM images. The schematics of each profile are also shown for clarity. All nanowires are shown in Fig. 2 are grown using *pre-growth conditions 1* described under the methods section. The <100> oriented nanowires in series (a) and (b), and panel (c) i are around 1 μm in length. The nanowires have similar morphology for most growth conditions and a 45 ° tilted side view SEM image of the standard sample is shown in the inset of Fig. 2a(iii). All <100> oriented nanowires showed the same facet profile for a given growth condition and large area top views of the same growths as those shown in Fig. 2 can be found in Additional file 1: Figure S1. As seen in the side view inset in Fig. 2a(iv), for the growth temperature of 475 °C, around a third of the vertically nucleated nanowires kinked towards a <111> direction at the top part of the nanowire (see Additional file 1: Figure S2). This is presumed to have taken place during the cooling down stage after growth with the depletion of In from the Au particle as shown in [26]. In this sample, the facets of the vertical [100] oriented segment are examined by focusing on the lower non-kinked part of the nanowire.

The temperature variation from 420 to 450 °C has drastically changed the facets from four {011} facets to four {001} facets via octagonal shape that comprises of both types of facets. Considering the similar nanowire heights of 1 μm, there is no significant difference in the tapering from 420 to 450 °C. The trend significantly changes at a growth temperature of 475 °C. Again, the height of the [100] oriented segment of these nanowires is 1 μm, which allows direct comparison of lateral growth by comparing the cross-sectional area. Radial growth of nanowires is generally kinetically limited [35]. This means radial growth is expected to increase with the temperature. Contrary to this expectation, the total lateral growth is less in this case. The lateral growth in the [01-1] and [0-11] directions is very small, although there is not much difference in the lateral growth in the [011] and [0-1-1] directions compared to lower growth temperatures. The side view of the nanowires reveals that some nanowires are thinner at the base (inset in Fig. 2a(iv)). The areas that has been grown earlier showing less lateral growth suggests that some surface decomposition and evaporation is taking place at 475°C. It should also be noted that these <100> nanowires are much more prone to thermal decomposition compared to <111> oriented nanowires of WZ or ZB phase. In a separate experiment, where WZ phase <111> nanowires and ZB <100> nanowires were heated to a higher temperature, it was seen that all <100> nanowires were completely decomposed during the temperature ramping from 450 to 650 °C, even under PH_3_ overpressure, while the <111> equivalent <0001> WZ nanowires still survived (Additional file 1: Figure S3). Here, a similar, lower level of decomposition could be taking place at the relatively low temperature of 475 °C, due to the low flow rate of PH_3_ and hence the lack of group V overprotection. Decomposition competing with the slow growth rate could also be the reason for the lack of nanowire growth at the growth temperature of 500 °C.

As discussed earlier, the inclined {011} facets show partial polarity and the partially B polar inclined (01-1) and (0-11) facets could be more susceptible to decomposition [32, 33]. This would lead to more competition from decomposition on the (01-1) and (0-11) facets compared to (011) and (0-1-1) facets, limiting lateral growth in the former facets compared to lower growth temperatures where decomposition is not present. This results in the highly elongated shape observed at 475 °C growth temperature.

Similarly, the V/III ratio should play a role in the resulting cross-section shape with a high V/III ratio promoting over-growth of partially A polar, off-cut (011) and (0-1-1) facets and hence, enhancing the asymmetry in the two perpendicular <011> directions. However, no such asymmetry is observed in the V/III range that is studied here (Fig. 2b series). One reason for this is the complete range (200 to 700) that was possible to be experimented within the reactor limitations while maintaining a high vertical yield, being relatively high in terms of V/III ratios generally used in MOVPE. Therefore, no obvious differences are seen in SEM analysis. Also, as the more prominent side facets that are dictated by the growth conditions is {001}, these asymmetries may already have been overgrown along with the bulk of the nanowire, to produce the more prominent symmetric {001} facets.

Increasing TMIn flow rate (and hence the growth rate) results in facets changing from {001} to {011} (Fig. 2c(i–ii)). Considering the longer length of the nanowires grown with higher TMIn flow rates (~ 1.5 and 2.5 μm for 12× and 20× flow rates, respectively), the tapering parameter (calculated as, (average nanowire width at the base–hemispherical NP diameter)/(2 × average nanowire length)) is actually decreasing with increasing flow rate, although the absolute lateral growth increases as seen in series (c) in Fig. 2. This reduction in tapering parameter with increasing precursor flow rate is expected in nanowires as the axial growth is mass transport limited and the radial growth is kinetically limited [35, 36]. Although, there was no clear evidence of current radial facet growth being kinetically limited, the increased mass transport limited axial growth rate with the precursor flow rate has contributed to the observed behaviour. The facets seen for the highest TMIn flow rate studied (~ 20×)) are interesting. The cross-section shape is roughly octagonal, yet it does not comprise of low surface energy and/or low index facets. These facets are complicated by the irregular micro-facets seen along with the side facets (see the facet on the front in the 45° titled view SEM inset in Fig. 2c(iii)). While the reason for the formation of these facets is not completely clear at this point, one possible reason could be the decrease in diffusion length of adatoms with their increase in supply [5, 37, 38]. In this case, the adatoms would not be able to migrate far enough to get incorporated at low energy sites or facets, but rather get incorporated closer to point of absorption forming higher energy micro-facets.

So far, it could be seen that most growth parameters used to grow the nanowires using *pre-growth conditions 1 *has resulted in symmetric {001} facets. The lowest growth temperature (420 °C) and higher (~ 10×) TMIn flow rate have yielded {011} type facets. However, these two conditions result in lower vertical yield (< 20 %) as shown in Additional file 1: Figure S1. Hence, *pre-growth conditions 2*, demonstrated by Wang *et al.* [26] was investigated to maintain a high vertical yield while carrying out growth under high TMIn flow rate, and achieve {011} type facets.

**Fig. 3:**
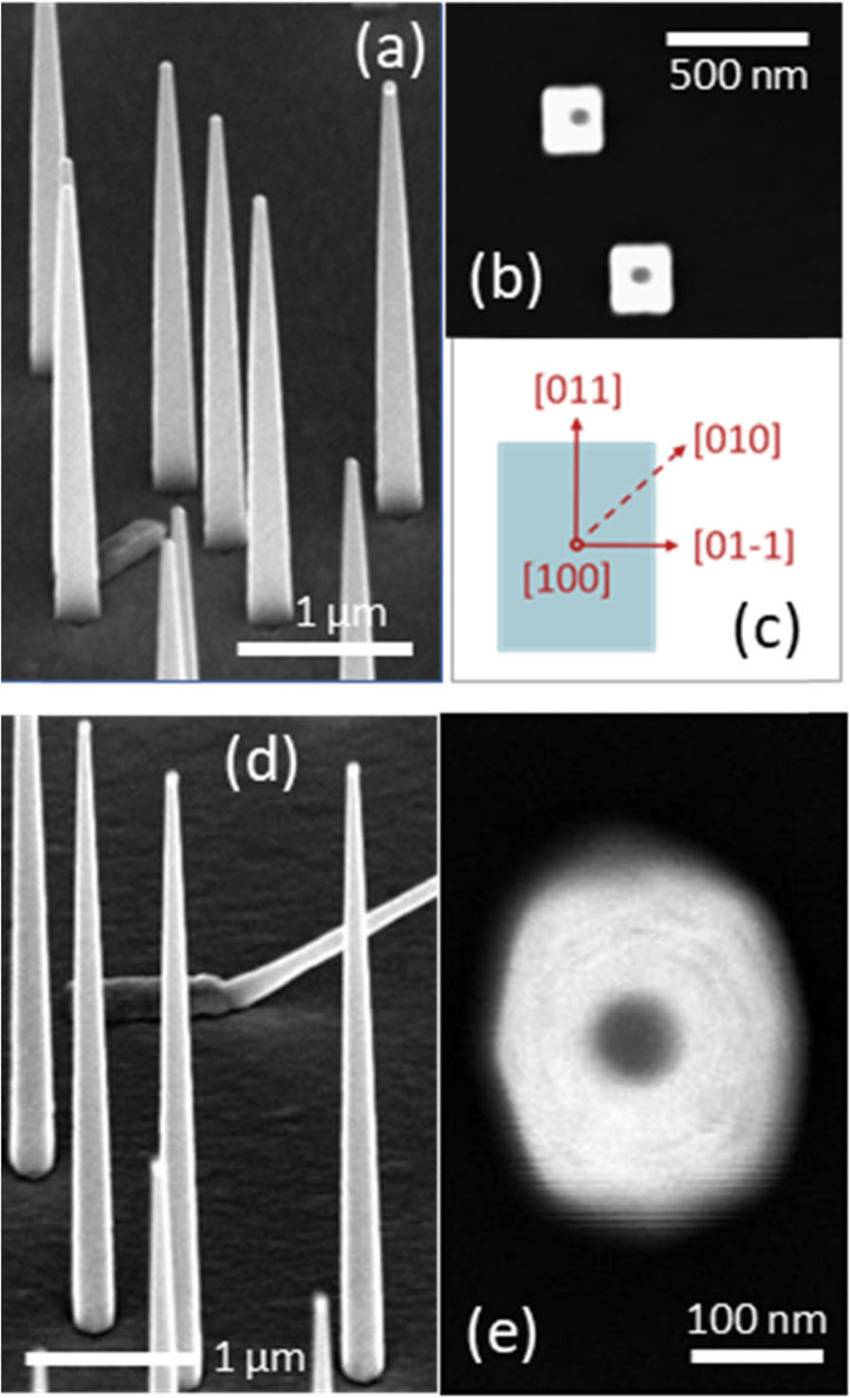
Facets of nanowires grown using TMIn pre-flow technique (**a**) 45˚ tilted SEM view of nanowires grown using the TMIn pre-flow technique and growth conditions given in Table 2 in methods section. (**b**) Top view of nanowires shown in (a). (**c**) Schematic showing the facet profile and the directions with respect to the substrate in (**b**). (**d**) 45˚ tilted SEM view of nanowires grown using TMIn pre-flow technique and 3 times higher flow rate as that of **(a**) and (**b**). (**e**) Top view of a nanowire from (**d**).

As shown in Fig. 3a, b, these growth conditions yielded ~ 65–80% vertical nanowires with <100> oriented nanowires having {011} side facets as expected. The cross-section is elongated in the [011]↔[0-1-1] directions due to a higher growth rate of the respective facets, resulting in a rectangular shape. It should be noted that similar growth conditions have resulted in {001} type side facets in the original study [26, 27], and this could be due to subtle differences such as reactor configuration and total flow. The TMIn flow rate could be further increased by three times, to a slightly higher value than that used in the growth shown in Fig. 2c(iii), without compromising the vertical yield (~ 72%) as shown in Fig. 3d. In this case, the particle pre-filling time was reduced by a factor of 3 in order to keep the In percentage in the particle almost the same at the time of nucleation. Large area top-view SEM images of the same growths as those shown in Fig. 3a, d can be found in Additional file 1: Figure S4. The facets of the resulting nanowires shown in Fig. 3e are similar to those previously seen for a very high TMIn flow rate in Fig. 2c(iii). This observation again confirms the argument that the facets are only dependent on the growth conditions and not the pre-growth conditions. In the following, these facets are further engineered to form low index combinations by in situ post-growth annealing.

After growth, the stability of the nanowire facet profiles is determined by surface energy and surface-to-volume ratio [23, 39]. The surface energy mainly depends on the type of facet, for example, {011} facets have lower surface energy compared to {001} facets [40, 41]. The surface-to-volume ratio, which is equal to circumference-to-area ratio (assuming constant nanowire height), is governed by the cross section shape; an octagonal cross section has a lower ratio compared to a square cross section. Annealing could provide thermal energy to overcome the kinetic energy barrier for surface migration of atoms [28], resulting in a facet profile that would minimise the total surface related energy with the optimum balance between the facet types and cross sectional shape. The amount of thermal energy that is supplied can be controlled by two annealing parameters, namely, temperature and time. These will in-turn control the volume of material that is migrated and the distance the atoms could migrate, and hence the resulting facet profiles of the nanowires.

As stated earlier, <100> nanowires cannot withstand high annealing temperatures, limiting the parameter range in terms of annealing temperatures. Hence, annealing time was used in this study in order to engineer the facets. Annealing was carried out directly after growth at 550 °C for durations between 20 s and 10 min under PH_3_ overpressure. It should be noted that surface migration also takes place during the temperature ramp-up from 450 °C growth temperature to 550 °C annealing temperature, which took about 210 s.

**Fig. 4:**
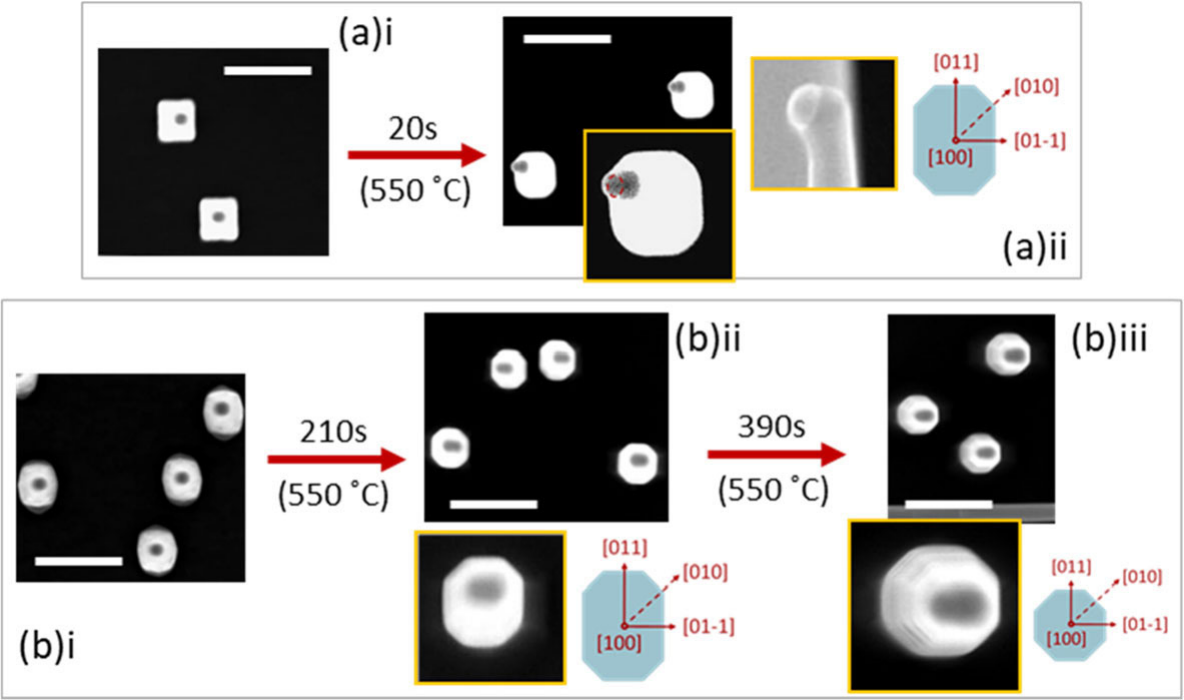
Facet engineering by post-growth annealing technique. Top view SEM images showing (**a**) facet evolution of nanowires with {011} facets after annealing for 20 s. (**b**) facet evolution of nanowires with high index facets after annealing for 210 and 600 s. Note that the apparent elongation of the Au particle seen in top view in (**a**) ii, (**b**) ii and (**b**) iii is due to the Au particle titling (as shown in the side view inset of a ii) with respect to the growth direction during annealing and/or cooling down. All scale bars are 500 nm

Figure 4a(ii), b(ii) show the resulting facets after annealing, for the nanowires shown in Fig. 3a, b and d, e for 20 and 210 s, respectively. In both cases, surface migration has taken place with the cross-section shape evolving into an elongated octagonal shape. This shape has a lower circumference-to-area ratio than the starting rectangular shape in the case of the series of nanowires shown in Fig. 4a. As for the nanowires shown in Fig. 4b, it could be seen that the high index facets have evolved into low index {001} and {011} facets that have lower surface energies. Existence of multiple intermediate steps in the rearrangement process could be the reason for the ten times longer annealing time required by the irregular faceted nanowires to reach elongated octagonal shape in Fig. 4b(i–ii), compared to those shown in Fig. 4a, where direct migration may have taken place. Further annealing of these facets for 6.5 min has completed the surface migration process resulting in a symmetric octagonal cross section. This shape evolution reduces the resultant total surface energy by reducing the surface-to-volume (or circumference-to-area) ratio, despite the shrinkage of the {011} facets and, formation and expansion of relatively higher energy {001} facets in the process.

Additional file 1: Table S1 extends Table 3 in the main manuscript to include experimental pre-growth, growth, and post-growth annealing parameters that result in cross-sectional shapes theoretically predicted for <100> nanowires, while maximising vertical yield.

**Fig. 5:**
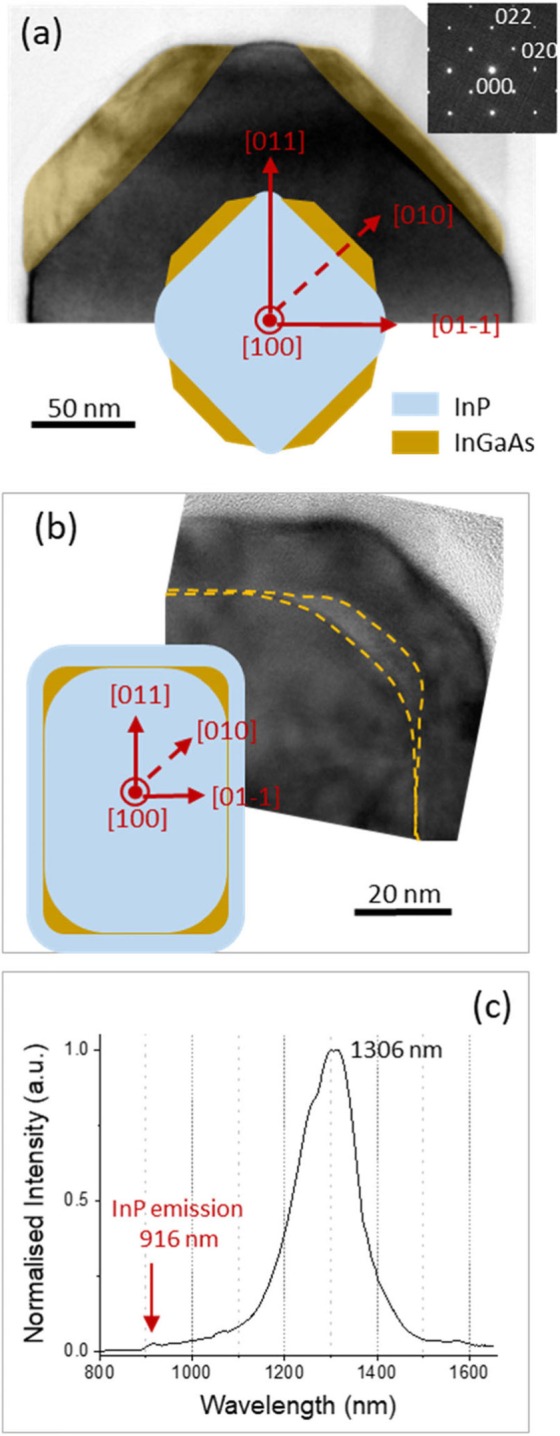
Structural and optical properties of heterostructure growth on [100] nanowire facets. Schematics and cross-section transmission electron microscopy (TEM) images of (**a**) separated InGaAs shell plates grown on a predominantly {001} faceted nanowire using a high flow rate. Inset shows the indexed diffraction pattern pertaining to the TEM image. (**b**) InGaAs quantum wires grown on an elongated octagonal cross-sectioned nanowire with smaller {001} facets, using a high flow rate. Insets show the schematics of the radial heterostructures. (**c**) Room temperature PL from a single nanowire from the same sample as (**b**), bright emission is observed from the QWR, while InP emission is visible as a very weak peak

As discussed in the introduction, non-uniform side facets can be exploited to create complex radial heterostructures. Figure 5a, b shows two examples of how continued preferential and anisotropic growth of subsequent layers could create unconventional radial heterostructures. It was seen in Fig. 2c(ii) and 3a–c, that higher precursor flow rate results in {011} facets. This means that the {001} facets grow faster under these conditions. Figure 5a shows an In_0.55_Ga_0.45_As layer grown on a [100] oriented InP nanowire core with larger {001} facets with a total group III flow rate of 1.23 × 10^−5^ mol/min, which is relatively high and comparable to those that yield {011} facets for the InP nanowires. Although the behaviour of facets of different materials may slightly vary, here too it is seen that the preferential and faster growth on {001} facets at high total precursor flow rates has resulted in the growth of separated InGaAs shell platelets on the {001} facets. Another InP layer grown with a moderate precursor flow rate could encapsulate the whole structure to form a quantum well (QW) plates that are separated from each other, which is in contrast to tubular radial QWs that are commonly observed in ZB <111> or WZ <0001> oriented nanowires [10, 42]. In addition to QWs, this concept will also allow the design and fabrication of four-sided devices on the side facets of the nanowires [7].

Figure 5b shows a similar InGaAs layer growth carried out on an InP nanowire core with elongated octagonal cross-sectional shape (type V in Additional file 1: Table S1) with smaller {001} facets. Here, the faster InGaAs growth on the {001} facets have resulted in the formation of quantum wires (QWRs) that run along the four {001} edges of the core nanowire. The subsequent InP layer grown with a medium TMIn flow rate of 6.75 × 10^−06^ mol/min has capped the growth, completing the barrier of the QWRs. Figure 5c shows the representative room temperature PL spectrum from a single nanowire from the same sample. Bright emission is observed at around 1.31 μm from the QWR, whereas InP core and barrier emission is barely visible, demonstrating the efficient carrier capture by the QWRs grown on the four {001} facets. The broadness in emission could be due to slight variations in size between the four QWRs and the subtle fluctuations in thickness along the length of the nanowire (see Additional file 1: Figure S5).

## Conclusions

The facets of the [100] oriented nanowires were engineered to obtain a variety of facet combinations resulting in cross-sectional shapes from square to the octagon. This was achieved while maintaining a high yield of vertical nanowires. Nanowire facets were solely dependent on the growth parameters and it was seen that slow growth rates resulted in {001} type facets, while fast growth rates mostly yielded {011} facets. Facets were further engineered by post-growth in situ annealing to form octagonal and elongated octagonal cross-section shapes comprising of a combination of {011} and {001} facets. The novel facets of [100] nanowires and their relative preferential growth were manipulated to demonstrate optically active novel types of radial heterostructures. These results should increase the interest in these nanowires grown on the industry standard (100) oriented substrates in a wide range of novel applications that are based on complex nanowire architectures.

## Additional File

**Additional file 1: Figure S1.** Low magnification top view SEM images of the same growths as those shown in Figure 2 in the main manuscript. **Figure S2.** A TEM image of a nanowire from the sample shown in Figure 2(a)iv. **Figure S3.** a 45 ° tilted view of the same nanowires sample as that in Figure 2(a)iv in the main manuscript and a sample where the reactor temperature was increased to 650 °C under PH_3_ flow and an InP shell was attempted to be grown at 650 °C after a nanowire core growth, **Figure S4.** Large area top view SEM images of the same growths as those shown in Figure 3 (a) and (d), respectively, **Table S1.** Summary of experimental pre-growth, growth and post-growth anneal parameters in order to achieve different facet profiles in Table 3**.** while maintaining a high vertical yield, **Figure S5.** TEM images of the QWRs viewed along the <001> zone axis.

## Data Availability

The datasets used and/or analysed during the current study are available from the corresponding author on reasonable request.

## Abbreviations

MOVPE: Metal organic vapour phase epitaxy
PL: Photoluminescence
QW: Quantum well
QWR: Quantum wire
SEM: Scanning electron microscopy
TEM: Transmission electron microscopy
TMIn: Trimethylindium
WZ: Wurtzite
ZB: Zincblende

## References

[1] Xu X, Servati P (2009). Facet-dependent electronic properties of hexagonal silicon nanowires under progressive hydroxylation and surface reconstruction. Nano Letters..

[2] Yang X, Shu H, Jin M, Liang P, Cao D, Li C (2014). Crystal facet effect on structural stability and electronic properties of wurtzite InP nanowires. Journal of Applied Physics..

[3] Sansoz F (2011). Surface faceting dependence of thermal transport in silicon nanowires. Nano Letters..

[4] Shin N, Chi M, Filler MA (2013). Sidewall morphology-dependent formation of multiple twins in Si nanowires. ACS Nano..

[5] Shen XQ, Nishinaga T (1995). Inter-surface diffusion of In on (111)A-(001) InAs nonplanar substrates in molecular beam epitaxy. Journal of Crystal Growth..

[6] Dimakis E, Jahn U, Ramsteiner M, Tahraoui A, Grandal J, Kong X (2014). Coaxial multishell (In,Ga)As/GaAs nanowires for near-infrared emission on Si substrates. Nano Letters..

[7] Tomioka K, Yoshimura M, Fukui T (2012). A III-V nanowire channel on silicon for high-performance vertical transistors. Nature..

[8] Paladugu M, Zou J, Guo Y-N, Zhang X, Joyce HJ, Gao Q (2008). Polarity driven formation of InAs/GaAs hierarchical nanowire heterostructures. Applied Physics Letters..

[9] Kempa TJ, Kim S-K, Day RW, Park H-G, Nocera DG, Lieber CM (2013). Facet-selective growth on nanowires yields multi-component nanostructures and photonic devices. Journal of the American Chemical Society..

[10] Qian F, Li Y, Gradecak S, Park H-G, Dong Y, Ding Y (2008). Multi-quantum-well nanowire heterostructures for wavelength-controlled lasers. Nat Mater..

[11] Guo Y-N, Burgess T, Gao Q, Tan HH, Jagadish C, Zou J (2013). Polarity-driven nonuniform composition in InGaAs nanowires. Nano Letters..

[12] Algra RE, Mr H, Verheijen MA, Zardo I, GGW I, WJP VE (2011). Crystal structure transfer in core/shell nanowires. Nano Letters..

[13] Arbiol J, Magen C, Becker P, Jacopin G, Chernikov A, Schafer S (2012). Self-assembled GaN quantum wires on GaN/AlN nanowire templates. Nanoscale..

[14] Fonseka HA, Velichko AV, Zhang Y, Gott JA, Davis GD, Beanland R et al (2019) Self-formed quantum wires and dots in GaAsP–GaAsP core–shell nanowires. Nano Letters 10.1021/acs.nanolett.9b01673 PMC7007271 31141668

[15] Svizhenko A, Leu PW, Cho K (2007). Effect of growth orientation and surface roughness on electron transport in silicon nanowires. Physical Review B..

[16] Schuurmans FM, Schonecker A, Eikelboom JA, Sinke WC (1996). editors. Crystal-orientation dependence of surface recombination velocity for silicon nitride passivated silicon wafers. Photovoltaic Specialists Conference, 1996. Conference Record of the Twenty Fifth IEEE.

[17] Kim S-K, Day RW, Cahoon JF, Kempa TJ, Song K-D, Park H-G (2012). Tuning light absorption in core/shell silicon nanowire photovoltaic devices through morphological design. Nano Letters..

[18] Foster AP, Bradley JP, Gardner K, Krysa AB, Royall B, Skolnick MS (2015). Linearly polarized emission from an embedded quantum dot using nanowire morphology control. Nano Letters..

[19] Saxena D, Wang F, Gao Q, Mokkapati S, Tan HH, Jagadish C (2015). Mode profiling of semiconductor nanowire lasers. Nano Letters..

[20] Vu TTT, Zehender T, Verheijen MA, Plissard SR, Immink GWG, Haverkort JEM (2013). High optical quality single crystal phase wurtzite and zincblende InP nanowires. Nanotechnology..

[21] Algra RE, Verheijen MA, Borgstrom MT, Feiner L-F, Immink G, van Enckevort WJP (2008). Twinning superlattices in indium phosphide nanowires. Nature..

[22] Fortuna SA, Li X (2010). Metal-catalyzed semiconductor nanowires: a review on the control of growth directions. Semiconductor Science and Technology..

[23] Zhang RQ, Lifshitz Y, Ma DDD, Zhao YL, Frauenheim T, Lee ST (2005). Structures and energetics of hydrogen-terminated silicon nanowire surfaces. The Journal of Chemical Physics..

[24] Fonseka HA, Caroff P, Wong-Leung J, Ameruddin AS, Tan HH, Jagadish C (2014). Nanowires grown on InP (100): growth directions, facets, crystal structures, and relative yield control. ACS Nano..

[25] Krishnamachari U, Borgstrom M, Ohlsson BJ, Panev N, Samuelson L, Seifert W (2004). Defect-free InP nanowires grown in [001] direction on InP (001). Applied Physics Letters..

[26] Wang J, Plissard SR, Verheijen MA, Feiner L-F, Cavalli A, Bakkers EPAM (2013). Reversible switching of InP nanowire growth direction by catalyst engineering. Nano Letters..

[27] Wang J, Plissard S, Hocevar M, Vu TTT, Zehender T, Immink GGW (2012). Position-controlled [100] InP nanowire arrays. Applied Physics Letters..

[28] Jiang N, Wong-Leung J, Joyce HJ, Gao Q, Tan HH, Jagadish C (2014). Understanding the true shape of Au-catalyzed GaAs nanowires. Nano Letters..

[29] Kayser O (1991). Selective growth of InP/GaInAs in LP-MOVPE and MOMBE/CBE. Journal of Crystal Growth..

[30] Zou J, Paladugu M, Wang H, Auchterlonie GJ, Guo Y-N, Kim Y (2007). Growth mechanism of truncated triangular III–V nanowires. Small..

[31] Shaw DW (1968). Effects of vapor composition on the growth rates of faceted gallium arsenide hole deposits. Journal of The Electrochemical Society..

[32] Lum WY, Clawson AR (1979). Thermal degradation of InP and its control in LPE growth. Journal of Applied Physics..

[33] Chu SNG, Jodlauk CM, Johnston WD (1983). Morphological study of thermal decomposition of InP surfaces. Journal of The Electrochemical Society..

[34] Carter WC, Roosen AR, Cahn JW, Taylor JE (1995). Shape evolution by surface diffusion and surface attachment limited kinetics on completely faceted surfaces. Acta Metallurgica et Materialia..

[35] Joyce HJ, Gao Q, Wong-Leung J, Kim Y, Tan HH, Jagadish C (2011). Tailoring GaAs, InAs, and InGaAs nanowires for optoelectronic device applications. IEEE Journal of Selected Topics in Quantum Electronics..

[36] Joyce HJ, Gao Q, Tan HH, Jagadish C, Kim Y, Fickenscher MA (2009). Unexpected benefits of rapid growth rate for III−V Nanowires. Nano Letters..

[37] Isu T, Hata M, Morishita Y, Nomura Y, Katayama Y (1991). Surface diffusion length during MBE and MOMBE measured from distribution of growth rates. Journal of Crystal Growth..

[38] Nishinaga T, Shen XQ, Kishimoto D (1996). Surface diffusion length of cation incorporation studied by microprobe-RHEED/SEM MBE. Journal of Crystal Growth..

[39] Wang N, Cai Y, Zhang RQ (2008). Growth of nanowires. Materials Science and Engineering: R: Reports..

[40] Sibirev NV, Timofeeva MA, Bol’shakov AD, Nazarenko MV, Dubrovskiĭ VG (2010). Surface energy and crystal structure of nanowhiskers of III–V semiconductor compounds. Phys Solid State..

[41] Liu QKK, Moll N, Scheffler M, Pehlke E (1999). Equilibrium shapes and energies of coherent strained InP islands. Physical Review B..

[42] Fonseka HA, Ameruddin AS, Caroff P, Tedeschi D, De Luca M, Mura F (2017). InP–InxGa1−xAs core-multi-shell nanowire quantum wells with tunable emission in the 1.3–1.55 μm wavelength range. Nanoscale..

