# Supplementary material for: Engineering the Side Facets of Vertical [100] Oriented InP Nanowires for Novel Radial Heterostructures
Source: Nanoscale Res Lett. 2019 Dec 30;14:399. doi: 10.1186/s11671-019-3177-6 (PMC6937364; doi:10.1186/s11671-019-3177-6)
Supplement: Supplementary file 1 — Additional file 1: Figure S1. Low magnification top view SEM images of the same growths as those shown in Figure 2 in the main manuscript. Figure S2. A TEM image of a nanowire from the sample shown in Figure 2(a)iv. Figure S3. a 45 ° tilted view of the same nanowires sample as that in Figure 2(a)iv in the main manuscript and a sample where the reactor temperature was increased to 650 °C under PH3 flow and an InP shell was attempted to be grown at 650 °C after a nanowire core growth, Figure S4. Large area top view SEM images of the same growths as those shown in Figure 3 (a) and (d), respectively, Table S1. Summary of experimental pre-growth, growth and post-growth anneal parameters in order to achieve different facet profiles in Table 3. while maintaining a high vertical yield, Figure S5. TEM images of the QWRs viewed along the <001> zone axis. [file 11671_2019_3177_MOESM1_ESM.pdf]

Supplementary Information for "Engineering the Side Facets of Vertical [100] Oriented InP Nanowires for Novel Radial Heterostructures"

H. Aruni Fonseka[1, 2,*], Philippe Caroff[1, ^], Yanan Guo[1, $], Ana M. Sanchez[2], Hark Hoe Tan[1] and Chennupati Jagadish[1]

[1]*Department of Electronic Materials Engineering, Research School of Physics and Engineering, The Australian National University, Canberra, ACT 2601, Australia*

[2]*Department of Physics, University of Warwick, Coventry CV4 7AL, United Kingdom.*

*Corresponding author: a.fonseka.1@warwick.ac.uk

^ Current Address: Microsoft Station Q at Delft University of Technology, 2600 GA Delft, Netherlands.

$ Current Address: Samsung Austin Semiconductors, 12100 Samsung Blvd, Austin, TX 78754, USA.

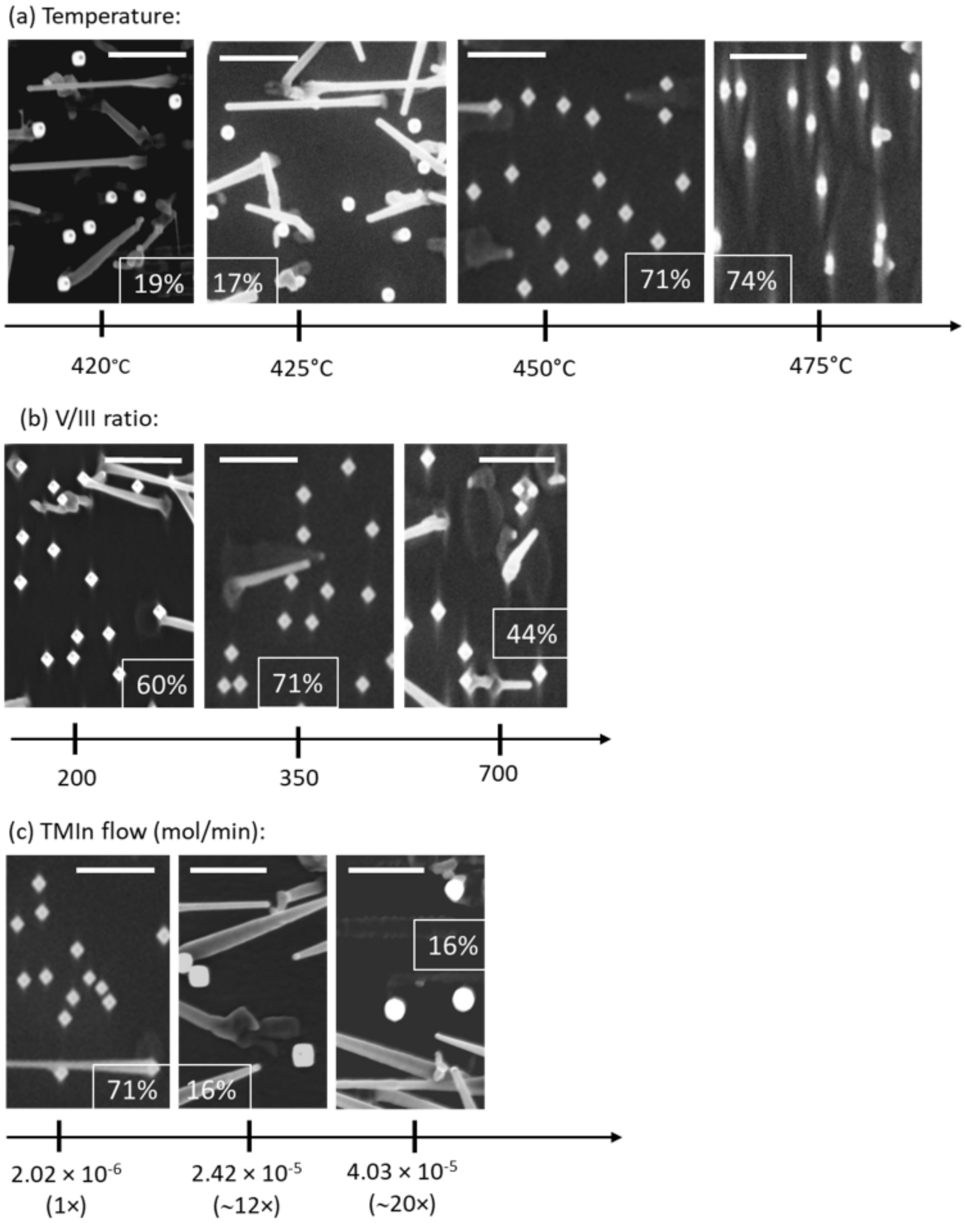

Figure S1: Low magnification top view SEM images of the same growths as those shown in Figure 2 in the main manuscript. The respective [100] vertical nanowire yields are given in the inset boxes. Scale bars are 500 nm.

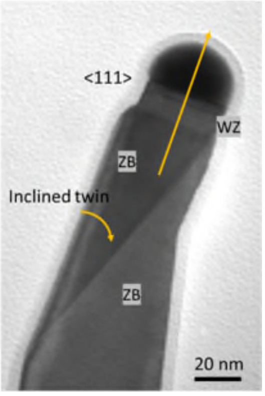

Figure S2: A TEM image of a nanowire from the sample shown in Figure 2(a)iv. The <111> oriented segment is of WZ crystal structure while the [100] segment is ZB.

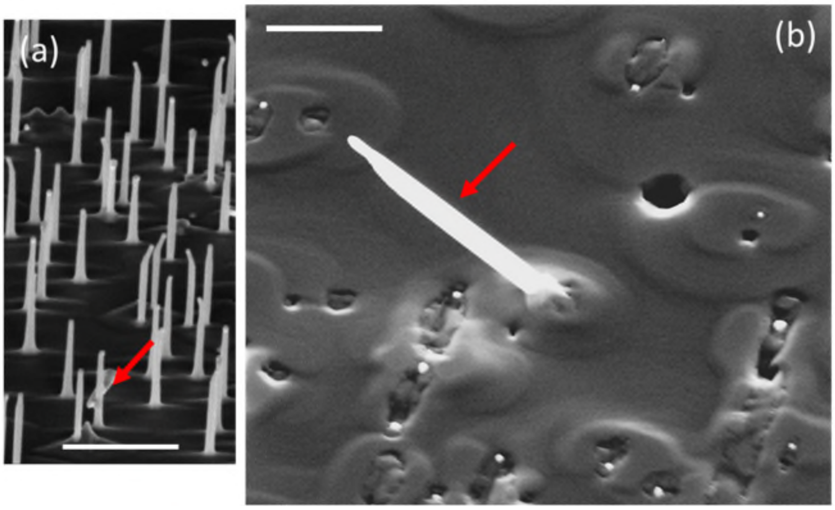

Figure S3: (a) 45 ° tilted view of the same nanowires sample as that shown in Figure 2 (a)iv in the main manuscript. (b) A sample where the reactor temperature was increased to 650 °C under PH_3 flow and an InP shell was attempted to be grown at 650 °C after a nanowire core growth similar to that in (a). The scale bars are 1 µm.

The non-vertical <111> oriented nanowires are marked by a red arrow in both images. It could be seen that only these nanowires are remaining in (b) while the <100> nanowires have decomposed. The catalyst particles left from the <100> nanowires are visible as bright dots in (b). Both, the differences in phase as well as the facets (and hence, their reactivity) could be possible reasons for the observed difference in the temperature tolerance of the <111> and <100> oriented nanowires.

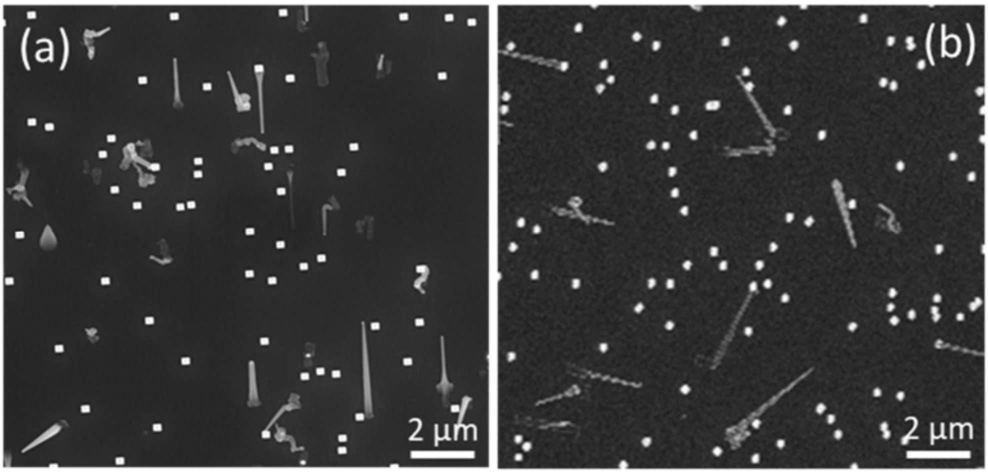

Figure S4: (a) and (b) are large area top view SEM images of the same growths as those shown in Figure 3 (a) and (d), respectively.

<table>
<tr><th></th><th>Facet combination</th><th>Cross sectional geometry</th><th></th><th>Pre-growth condition, growth parameter varied from standard sample & post-growth anneal condition</th><th>SEM</th><th>Type</th></tr>
<tr><td>1</td><td>Four {001} facets</td><td>Square</td><td>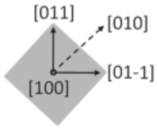</td><td>Pre-growth condition 1
Standard sample growth</td><td>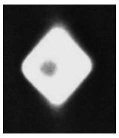</td><td>I</td></tr>
<tr><td rowspan="2">2</td><td rowspan="2">Four {011} facets</td><td>Square</td><td>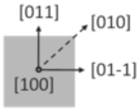</td><td>Could not be obtained, as vertical growth requires high V/III ratio</td><td></td><td>II</td></tr>
<tr><td>Rectangle</td><td>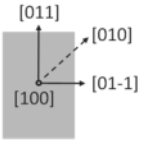</td><td>Pre-growth condition 2
Standard sample growth</td><td>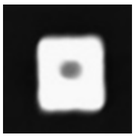</td><td>III</td></tr>
<tr><td rowspan="4">3</td><td rowspan="4">Combination of {001} and {011}</td><td>Perfect octagon</td><td>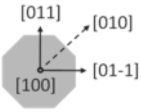</td><td>Pre-growth condition 2, but @ 3× TMIn flow rate
TMIn flow rate: 3×
Post-growth annealing: 10 min @ 550˚C</td><td>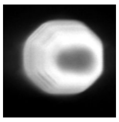</td><td>IV</td></tr>
<tr><td>Elongated octagon</td><td>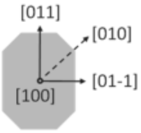</td><td>Pre-growth condition 2
Standard sample growth
Post-growth annealing: 20 s @ 550˚C
OR
Pre-growth condition 2, but @ 3× TMIn flow rate
TMIn flow rate: 3×
Post-growth annealing: 210 s @ 550˚C</td><td>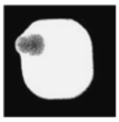
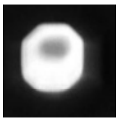</td><td>V</td></tr>
<tr><td rowspan="2">Hexagon</td><td rowspan="2">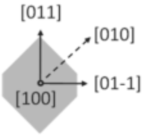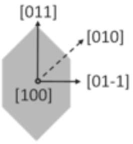</td><td rowspan="2">Pre-growth condition 1
Growth temperature: 475˚C

Note: Most nanowires contain a short kinked segment on top</td><td rowspan="2">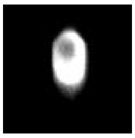</td><td>VI</td></tr>
<tr><td>VII</td></tr>
</table>

Table S1: Summary of experimental pre-growth, growth and post-growth anneal parameters in order to achieve different facet profiles in Table 3, while maintaining a high vertical yield

It should be noted that some nanowires may change the relative proportion of the facet sizes and hence alter the cross-sectional shape along its length. However, the general profile remains.

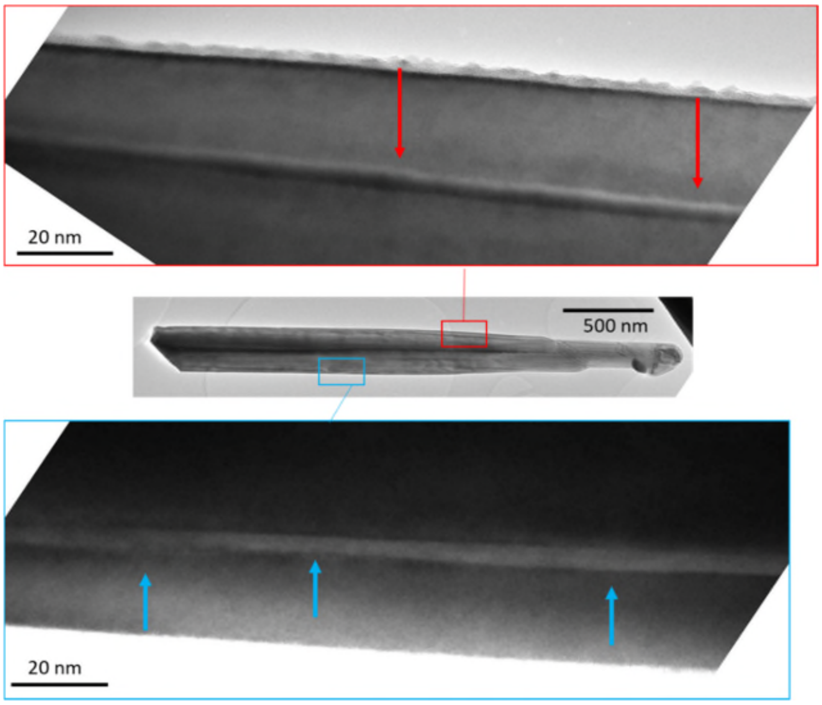

Figure S5: TEM images of the QWRs viewed along the <001> zone axis. The arrows indicate the variations in thickness of the QWRs along the nanowire.
